# Comparative analysis of macrophage associated vectors for use in genetic vaccine

**DOI:** 10.1186/1479-0556-9-10

**Published:** 2011-06-18

**Authors:** Mohammad Feraz Ahsan, Milind M Gore

**Affiliations:** 1 National Institute of Virology, Pashan Campus, 130/1, Sus Road, Pashan, Pune, 411021, India

## Abstract

**Background:**

Antigen presentation by non professional antigen presenting cells (APC) can lead to anergy. In genetic vaccines, targeting the macrophages and APC for efficient antigen presentation might lead to balanced immune response. One such approach is to incorporate APC specific promoter in the vector to be used.

**Methods:**

Three promoters known to be active in macrophage were selected and cloned in mammalian expressing vector (pAcGFP1-N1) to reconstruct (pAcGFP-MS), (pAcGFP-EMR) and (pAcGFP-B5I) with macrosialin, EmrI and Beta-5 Integrin promoters respectively. As a positive control (pAcGFP-CMV) was used with CMV promoter and promoterless vector (pAcGFP-NIX) which served as a negative control. GFP gene was used as readout under the control of each of the promoter. The expression of GFP was analyzed on macrophage and non-macrophage cell lines using Flow cytometry and qRT-PCR with TaqMan probe chemistries.

**Results:**

All the promoters in question were dominant to macrophage lineage cell lines as observed by fluorescence, Western blot and quantitative RT-PCR. The activity of macrosialin was significantly higher than other macrophage promoters. CMV promoter showed 1.83 times higher activity in macrophage cell lines. The expression of GFP driven by macrosialin promoter after 24 hours was 4.40 times higher in macrophage derived cell lines in comparison with non macrophage cell lines.

**Conclusions:**

Based on this study, macrosialin promoter can be utilized for targeting macrophage dominant expression. *In vivo *study needs to be carried out for its utility as a vaccine candidate.

## Background

DNA vaccination, wherein plasmid DNA encoding the desired antigen is inoculated in the host is thought to be one of the best approaches to combat several challenging diseases. The DNA thus elicits both the arms of immune response following *in vivo *expression of the antigen [1]. It has been endeavoured for the treatment of autoimmunity [2], cancer [3], allergic diseases [4] bacterial infections [5] and viral diseases [6]. Several strategies have been proposed to improve the efficacy of DNA vaccine, such as the use of liposomes [7], inclusion of CpG motif [8], administration of plasmid expressing costimulatory molecules and cytokines [9], exploring different routes of administration of vaccine [10-12] and targeting the vaccine to specific cells [13]. Targeting of DNA to endosomal/lysosomal compartment has also been explored to enhance the immune response [14].

Successful immune response requires engagement of T cell receptor with MHC-peptide on professional antigen presenting cell (APC) as a first signal. Simultaneously second signal in the form of various costimulatory molecule engagement is necessary for sustained immune response. Failure to have this second signal may lead to reduced immune response or even anergy [15]. In DNA vaccines, expression of antigen in non APC cells might lead to such an outcome. In order to achieve the APC specific expression is to target the antigen expression in professional APC. For the treatment of HIV-1, APC have been targeted through *ex vivo *priming by expressed antigen and reinoculation [16]. Another approach is to target the expression to APC without expression in non APC cells, which could be achieved by using promoters active only in APC [17]. Dendritic cell as an APC has gained major attention over macrophage and B cells as a potent cell in priming and stimulating naïve T cells. Langerhans cells have been targeted by Dectin-2 promoter [18]. Lentiviral vectors were also studied to deliver the gene into APCs [19]. CD11c promoter was widely studied as a DC selective promoter [20].

Though DC specific promoter has shown promising results, it also has some inconsistencies. In an immunization study, DC restricted DNA vaccine could not generate either humoral or cellular response and the role of B cell in cross presentation of antigen was thought to be responsible [21]. Moreover, a study has reported that targeting of DC was insufficient to optimally induce T cell immunity and the role of non-DC needs to be explored for sustained effector functions during DNA vaccination [22]. Hence the role of other professional APC (Macrophage and B-cells) as a target cell for DNA vaccine could not be ignored. It has been shown that macrophages are potent enough to stimulate naïve CD8 T cells to proliferate and mature [23]. *In vitro *studies have shown that macrophages are as good as DC in cross presentation of antigen [24], B cells have been shown to prime naïve CD4 T cells [25]. Thus there is a need to explore promoters which could be active also in other cells of APC and just not a single population.

The current study is aimed at *ex vivo *evaluation with a comparative account of macrophage dominant promoters in reference to widely used CMV promoter. Such promoters were selected on the basis of their expression profiles and association with activation following antigen encounter. GFP based reporter system was exploited due to its comparable sensitivity as the luciferase system and can be used to monitor expression of cells with low transfection efficiency [26]. Such expression studies of DNA vaccine to limited cells could also help us to improve the safety in clinical implication.

## Methods

### Cloning

Plasmid used in the study was pAcGFP1-N1 (Clonetech, Takara, USA) which has CMV as an immediate early promoter and GFP as a reporter (pAcGFP-CMV). Promoters were selected based on the published data. For the construction of various promoter constructs, RAW 264.7 cell line was used for genomic DNA isolation (Tri-reagent, MRC) and subsequently used to amplify promoters from sequences [GenBank: AF039399], [GenBank: AJ295275] and [GenBank: AF022111] for macrosialin, Emr I and Beta-5 Integrin respectively using primers (Table 1). The restriction sites for insertion in the plasmid were included in primers as indicated. Respective amplicons were cloned in StrataClone™ PCR Cloning kit (Stratagene,USA) and digested with the sets of restriction enzymes (Table 1).

**Table 1:** Primers used for cloning to amplify promoters with underlined restriction sites.

Promoter (Constructs)	Primer Sequence (5' → 3')	Restriction site
Macrosialin (pAcGFP-MS)	F-TATTAATGACCAAATCTACAGGGAGAACCC	VspI/Eco47III
		
	R-AGCGCTAGATGCTCAGACCAGCTA	

EMR-1 (pAcGFP-EMR)	F-TCATATGGAATTCTTTGTTTAGGTCTGTATGC	NdeI/Eco47III
		
	R-TAGCGCTTACTGTGGCAGTCATTCA	

Beta-5 Integrin (pAcGFP-B5I)	F-CCGATTAATATTCAAACGCCTTAGGTAGGTTT	VspI/Eco47III
		
	R-AGCGCTTCTACTCTCGGAGACCCT	

F: Forward Primer, R: Reverse Primer, Underlined sequences are the restriction enzyme sites

pAcGFP-CMV was digested with VspI/Eco47III restriction enzymes to excise CMV promoter. The digested products were cloned to reconstruct the respective vector. Promoterless vector (pAcGFP-NIX) for negative control was created by excision of CMV promoter using VspI/Eco47III sites and self ligated after klenow treatment. All reconstructed clones were confirmed through restriction analysis and sequencing.

**Figure 1:**
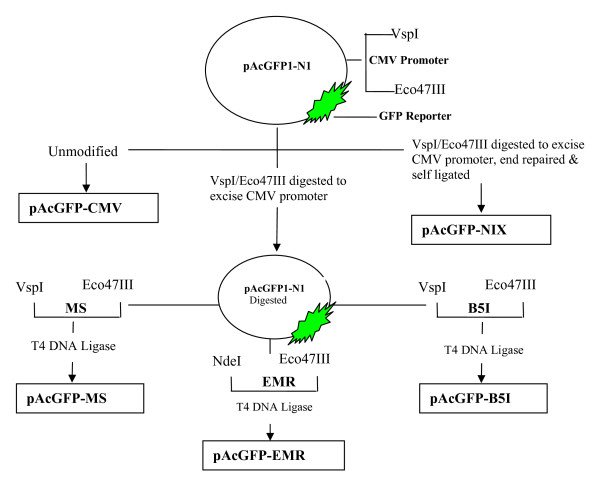
**Schematic representation of reconstructed promoters constructs with GFP as a reporter gene**.

Plasmids were prepared using Qiagen Maxiprep, according to manufacturer's protocol. The quantity and quality of plasmids was assessed using nanodrop by light absorption at 260/280 nm ratio and by 1% agarose gel electrophoresis. All the plasmids were dissolved in nuclease free water. The overall strategy of cloning and construction of plasmids with specific promoters is shown in Figure 1.

### Cell culture

RAW 264.7 (National Center for Cell Sciences, Pune, India) was maintained in high glucose DMEM with 10% fetal bovine serum (FBS) (Gibco, USA). This cell line was selected as a model to study expression in mouse macrophage cell lines [27]. L929 was obtained from the American Type Culture Collection (Rockville, MD) and maintained in MEM (Sigma) with 10% FBS. This cell line served as a modality to study expression in non-macrophage cell [28]. All cultures were incubated at 37°C, 5% CO_2 _in humidified environment. Antibiotic free media were used during transfection and for regular maintenance of cells.

### Transfection

Newly constructed pAcGFP-CMV, pAcGFP-MS, pAcGFP-EMR, pAcGFP-B5I, pAcGFP-NIX plasmids were used for transient transfection experiment. Transfections were performed using Lipofecatmine ™ 2000 (Invitrogen, USA). RAW264.7 and L929 cells were harvested and seeded in 6 well plates (3 × 10^5 ^cells/well). The plate was then incubated for 16 hours and after reaching confluence was transfected using 2 μg of each plasmid with 2 μl Lipofectamine 2000 as per manufacturer's protocol. Opti-MEM^® ^I reduced serum media (Invitrogen, USA) was used as a medium for transfection. Negative control was used both for Lipofectamine 2000 and plasmid DNA.

### Western blot

Expression of GFP protein was analysed by Western blot using standard protocols. Briefly, 24 hours after transfection with different DNA constructs encoding GFP, RAW 264.7 cells were harvested, washed twice with PBS, mixed with an equal volume of 2 ×loading buffer and boiled for 10 min. Proteins form 50 μg of cell lysate were separated onto a discontinuous SDS-polyarylamide gel with 5% stacking gel and 12% separating gel and transferred to a nitrocellulose membrane (Amersham Biosciences, USA). The membrane was blocked by 5% skimmed milk powder in PBS and then incubated with anti-GFP Ab (1:1000, Clontech) followed by goat anti-mouse IgG-HRP conjugate (1:5000, Sigma). Bands were visualized with substrate solution containing diaminobenzidine tetrahydrochloride and H_2_O_2 _solution.

### Fluorescent Microscopy

Both RAW 264.7 and L929 cells were monitored for GFP fluorescence at 6, 12, 24, 36 and 48 hours post transfection under UV microscope (Nikon eclipse Ti). The setting of microscope and camera was constant throughout, so as to get the semi-quantitative analysis. The photograph was captured with following settings: Resolution- Fast; Focus-640 × 480; Quality-2560 × 1920; Mode-manual exposure; Exposure-800 ms; Gain-1.20×; Objective-20×; Contrast- high. The software used for the analysis was: NIS-Elements BR version 3.1

### Flow cytometry

After transfection at different time points, cells were harvested by trypsinization, washed twice with PBS and suspended in FACS buffer (PBS + 2% FBS and 0.1% sodium azide). All samples were analysed using FACS Calibur (Becton Dickinson) and data were analysed using CellQuest Pro (Becton Dickinson) software. 10,000 events were used for analysis. GFP was excited through argon LASER and fluorescence was captured in FL1 channel by using 530/30 nm bandpass filter. The debris and dead cells were excluded using FSC and SSC parameters. Mean fluorescence was used to evaluate the level of GFP expression above the threshold level of autofluorescence of non-transfected control cells. For each assay three independent transfections were performed and mean fluorescence with ± SEM was used for analysis.

### Standardization of quantitative RT-PCR for detection of GFP mRNA

#### Primer and probe design

**Figure 2:**
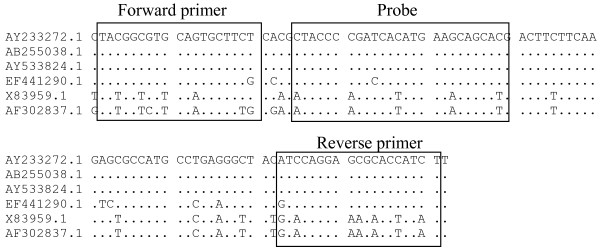
**Sequence alignment of GFP variants in GenBank showing the location of primers and probe**. GFP sequences were selected from data bank and aligned using MEGA4 software. The references of sequences are mentioned with the Accession number of GenBank. The sequence used for the primer and probe design was: Accession number-AY233272, GI-34421677.

Selected GFP sequences available in the GenBank were aligned using MEGA4 software [29]. Primers and probe were designed using Primer Express software™ 3.0 (Applied Biosystems International, Foster City, CA) (Figure 2). Primers and probe were picked from GFP sequence [GenBank: AY233272] nt. 196-295 with amplicon size of 100 bp. The probe was labelled with FAM (5-carboxyfluorescein) at the 5'end and BHQ-1 (Black hole quencher 1) at 3'end.

#### Preparation of RNA standard for the qRT-PCR

The 187 bp region was amplified using primer sets (Table 2, Cloning) from vector pAcGFP1-N1 and cloned into the pGEM^® ^-T Easy cloning vector (Promega Corporation, Madison, USA). The orientation of the insert was confirmed by sequencing. Plasmid was linearised by SpeI restriction enzyme. Target sequence was transcribed *in vitro*, DNAase treated and purified by MEGAscript^® ^kit (Ambion, USA) as per manufacturer's instructions. The RNA was quantified by spectrophotometry. The copy numbers of the RNA was calculated based on the concentration and its molecular weight. Ten fold serial dilutions of RNA from 10^2 ^to 10^10 ^copies per reaction were used as standard in all qRT-PCRs.

**Table 2:** Nucleotide sequence of primers and probe used in pGEM-T Easy cloning and qRT-PCR assay.

Assay	Primer/Probe	Sequence (5'→ 3')	Nucleotide positions
qRT-PCR	Forward Primer	TACGGCGTGCAGTGCTTCT	196-214

	Reverse Primer	AGATGGTGCGCTCCTGGAT	277-295

	TaqMan Probe	CTACCCCGATCACATGAAGCAGCACG	219-244

Cloning	Forward Primer	AAGTTCATCTGCACCAC	133-149

	Reverse Primer	TGTAGTTGCCGTCATCCT	302-319

#### qRT-PCR

After the desired period of post transfection, total RNA was extracted from the cell pellet of RAW 264.7 and L929 cells using RNEasy kit (Qiagen, Valencia, CA) and DNAse treated as per the manufacturer's protocol. RNA was eluted in 50 μl RNAse-free water and stored at -80°C. 5 μl (300 ng) of total RNA was used for all qRT-PCR for transfected cells. All reactions were carried out along with standards. The assay was run in triplicates in Rotor-Gene 3000 ™ (Corbett Research, Sydney, Australia) with the following thermal steps, RT at 50°C for 15 min, initial denaturation at 95°C for 2 min, 45 cycles of denaturation at 95°C for 15 sec and annealing with extension at 60°C for 30 sec. Fluorescence data were collected at the end of each cycle. Each reaction comprised no template control (NTC), cell control and cells treated with plasmid without transfectant. Primers and probe were used from a range of 100 to 600 nM for optimum concentration. CT values were recorded each time. 200 nM of forward and reverse primer with 100 nM of probe were found to be optimal for one step qRT-PCR in 25 μL final reaction volume. Optimised concentration of primer and probes were used to detect the copy number of *in vitro *transcribed RNA (IVT-RNA).

#### Statistics

All the data obtained through Flow cytometry or qRT-PCR was analysed for statistical significance using General Linear model, Tukey's comparison test. Analysis was performed using SPSS version 11 software.

## Results

### Selection of promoter

Following promoters were selected for the studies based on their known expression profiles. Macrosialin is a glycoprotein expressed specifically in murine monocytes and macrophages, and to a lesser extent by DC [30-32]. Macrosialin is murine homologue of CD 68 sharing 80% similarity [32]. Emr-1 (EMR) promoter is reported to control its gene expression mainly in macrophages [33-35]. The human orthologue of EmrI is EMRI. The promoter of EmrI and EMRI share 60% identity and is with purine rich conserved region. Its gene product has also served as a marker for macrophage population in many immunohistological studies [36]. Beta-5 Integrin promoter is expressed in macrophages and osteoblasts [37,38]. Integrin belongs to the family of type I transmembrane glycoprotein. It helps in cell migration, proliferation and differentiation. As a positive control we chose immediate early promoter of cytomegalovirus (CMV) which is widely used and is strong enough to drive constitutive expression in all cell types. As a negative control promoterless vector was constructed. This vector though has GFP as a reporter gene but is devoid of any promoter. All the selected promoters except CMV are TATA-less promoters and have PU.1 as a transcription factor which assembles the transcription machinery on myeloid promoters.

### Promoter amplification from genomic DNA and expression studies of various promoter constructs

**Figure 3:**
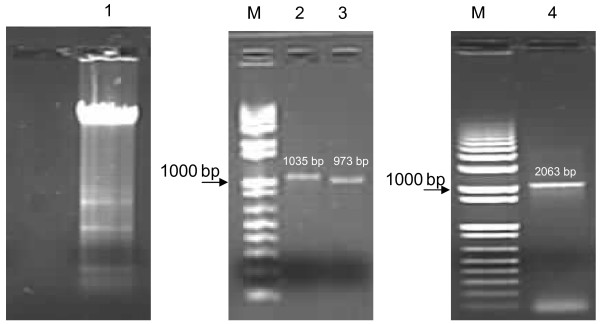
**PCR analysis of amplified promoters**. M: 1 Kb+ Ladder (Invitrogen); 2: Macrosialin; 3: Beta-5 Integrin; 4: EMR1 are the respective amplicons of promoters documented in 1% Agarose gel in TAE buffer.

**Figure 4:**
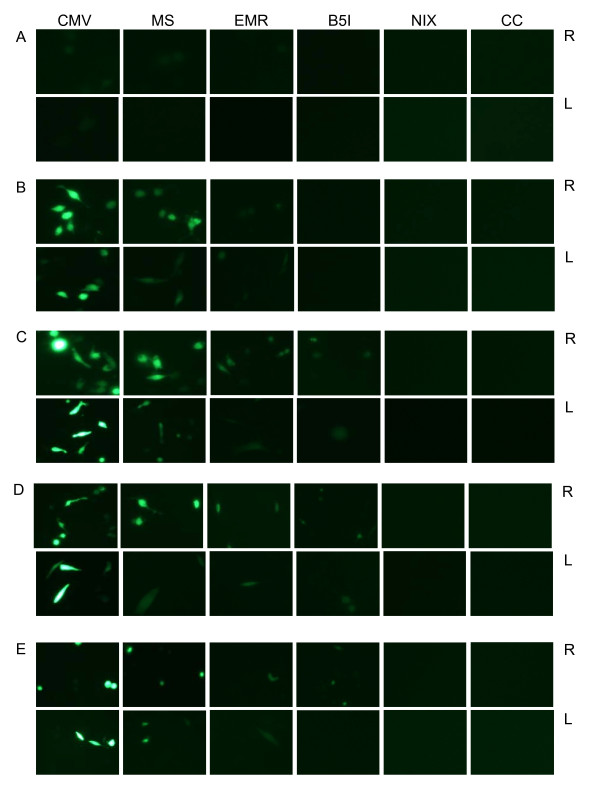
**Fluorescent Microscopy pictures of cells transfected with respective plasmid**. Expression of GFP in transfected RAW 264.7 (R) and L929 (L) cells at different time points as: A-6 hrs, B-12 hrs, C-24 hrs, D-36 hrs and E-48 hrs. The constructs for the transfected cells are mentioned at the top which follows throughout the respective column followed by (pAcGFP-). CC represents cell control.

Promoter sequences were amplified from RAW264.7 cells using Tri-reagent (MRC) and PCR. Amplicons obtained are shown in Figure 3. These were further used for cloning after sequence confirmation. Expression of GFP with different promoter constructs was analysed by fluorescent microscopy. Strong GFP expression was detected with pAcGFP-CMV in RAW264.7 and L929 cells, in contrast no GFP expression was observed with pAcGFP-NIX or Untransfected cells at any time point of studies (Figure 4). Figure shows representative pictures taken at different time point for each cell type (06-48 hrs following transfection) (Figure 4, A, B, C, D and 4E). Fluorescence of cells transfected with pAcGFP-MS was significantly higher than other modified constructs expressing GFP. The difference in fluorescence intensities were observed when the same constructs were used for RAW 264.7 and L929 cells. As expected non macrophage cell line L929 showed lesser expression of GFP driven by APC promoters.

### Western blot

**Figure 5:**
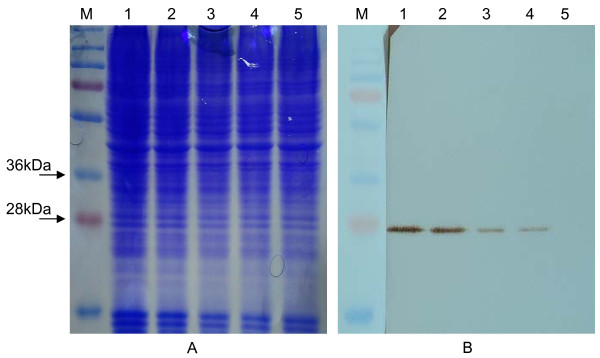
**PAGE/Western blot Analysis**. (A) 12% SDS-PAGE gel (B) Western blot analysis of the total cell lysates of the RAW 264.7 cells. M: PageRuler™ (Fermentas); 1: pAcGFP-CMV; 2: pAcGFP-MS; 3: pAcGFP-EMR; 4: pAcGFP-B5I; 5: pAcGFP-NIX. The blot shows expressed GFP protein from different constructs after 24 hours of transfection.

The transfected RAW 264.7 cell lysates prepared after 24 hours post transfection were subjected to Western blot analysis. The anti-GFP monoclonal antibody reacted specifically with GFP protein of ~27 kDa. Negative control did not show detectable levels of GFP. Strong expression of GFP under CMV promoter, served as a positive control (Figure 5, A and 5B).

### Flow cytometry analysis of GFP with different promoter constructs

**Figure 6:**
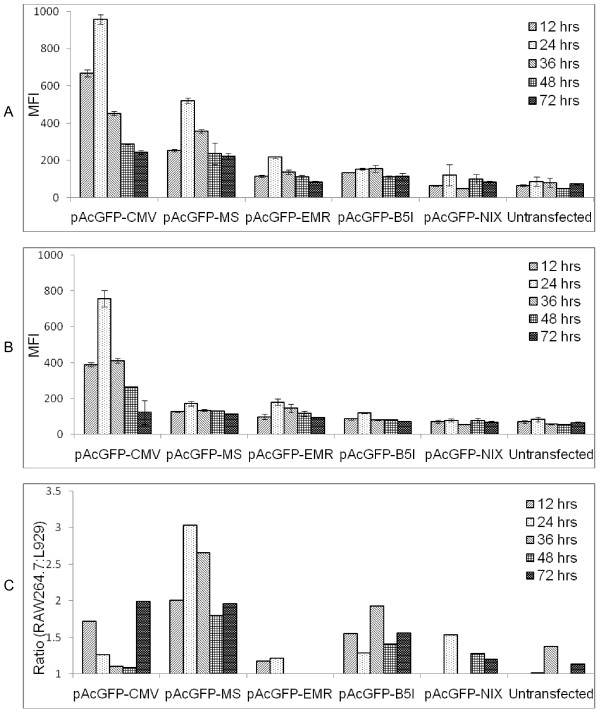
**Flow cytometry analysis**. Mean fluorescence of cells of different constructs transfected in (A) RAW264.7, (B) L929 cells. (C) Ratio of (RAW264.7/L929) were determined as an expression of macrophage specificity. The activity was measured at various time points. The average and SEM shown are from three independent assays. For ststistical analysis, General Linear Model (GLM), Tukey's comparison test was performed to compare the significance difference on fluorescence level amongst transfected plasmid.

Preliminary screening was performed using fluorescent microscope, green fluorescence was observed in cells transfected with respective constructs, confirming the successful protein expression. Precise specificity and strength of the promoter constructs were evaluated by Flow cytometry through transient transfection in RAW264.7 and L929 cells. MFI of pAcGFP-CMV construct after 24 hours was 11 fold in RAW 264.7 and 8.8 fold in L929 cells over that of Untransfected cells, whereas 6 fold and 2 fold in RAW264.7 and L929 cells respectively for pAcGFP-MS (Figure 6, A and 6B). The MFI of cells transfected with different constructs was significantly higher (p <0.05) when compared with Untransfected cells. No significant difference was observed between pAcGFP-NIX and Untransfected cells at any time point of studies. The differential level of expression of pAcGFP-MS when compared in RAW 264.7 and L929 cells, was found to be highly significant up to 36 hours. Similarly it was significant for pAcGFP-B5I up to 48 hours and non-significant for pAcGFP-EMR at all time points. For the comparative account of promoter specificity we have also used ratio of promoter activity in macrophage to that of non macrophage cells (Figure 6C). Among the promoters under study macrosialin promoter drove the high expression of reporter gene and conferred the highest myeloid specificity. This ratio could not be taken as absolute values due to variance in transfection efficiency in both the cell lines, rather it rendered a useful index of specificity.

Studies were also carried out using P388D1 and Vero cells as a macrophage and non macrophage cells respectively. Fluorescent microscopy showed the same trend of expression with different constructs (data not shown). It was difficult to transfect P388D1 cell line when the protocol mentioned above for the other cells were followed. The efficiency of transfection was very low. Increasing the Lipofectamine 2000 concentration increased the efficiency slightly. The expression levels directed by the promoters were highest after 24 hours. Intensity of GFP expression through CMV promoter was the highest followed by macrosialin and the other two promoters, following the same trend of expression as that of RAW264.7. Similarly, expression level in Vero cells was same as L929 cells, however, they got transfected with ease. Hence we carried out our further study on RAW264.7 and L929 cells.

### Quantification of GFP in transfected cells

**Figure 7:**
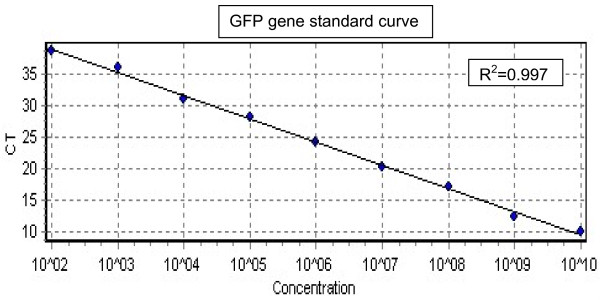
**Standard curve plot of log10 diluted *in vitro *transcribed RNA for GFP**.

The assay was sensitive enough to detect <100 copies of IVT-RNA (CT = 38.59). Linear correlation value in CT values obtained over the range of IVT-RNA per reaction was (R^2 ^= 0.99), when 10^2 ^to 10^10 ^copies were used. The assay did not amplify any non specific sequence from cellular RNA of cells used. There were clean bands of amplicons when observed in agarose gel electrophoresis. To check the reproducibility of the assay the standards were run on six different days and similar CT values were found for the given inputs of IVT-RNA. The data is the representative of the test (Figure 7).

**Figure 8:**
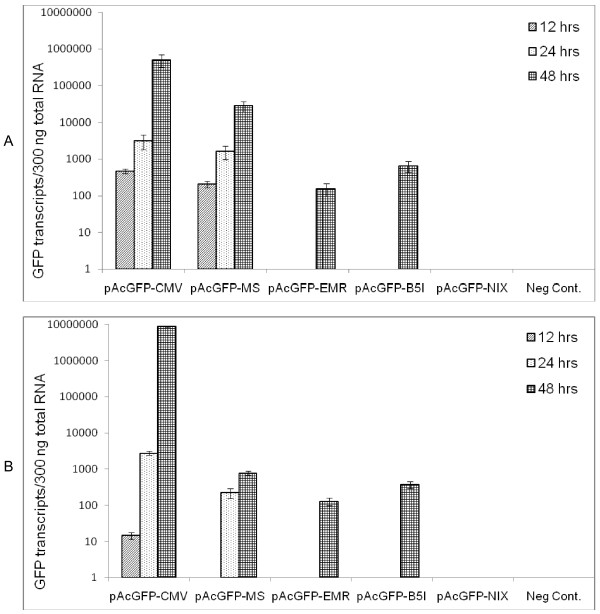
**Transcript profiling of GFP**. Transcript profiling of RAW264.7 (A) and L929 (B) cells transfected with different promoter constructs at the given time interval.

RNA was quantified post transfection after 12, 24 and 48 hours. It was observed that GFP in construct with CMV promoter was highly expressed in both RAW 264.7 and L929 cells (5.07^5 vs 8.94^6). The construct with macrosialin promoter showed >36 fold copy numbers in RAW264.7 cells in comparison to L929 cells at the end of 48 hrs. Data represented here is from analysis of three independent transfection assays with ±SEM. One way ANOVA, Tukey's comparison test was performed to compare the GFP transcripts in cells transfected with different construct. pAcGFP-CMV and pAcGFP-MS has a significantly higher number of GFP transcripts compared with Untransfected or pAcGFP-NIX construct (P <0.05). We get no amplification in Untransfected and pAcGFP-NIX (Figure 8, A and 8B)

## Discussion

The promoters of viruses are widely used in many mammalian expression vectors due to their strong activity in large variety of cells. CMV promoter has been of choice because of high level of constitutive expression in several mammalian cell lines [39]. Constitutive expression of gene could be suitable for gene therapy or certain applications [40]. However importance of using lineage specific promoter in DNA vaccine to limit gene expression to the target cells is of paramount importance, not only as an adjuvant [41] but also as a safety concern [42].

In the present study, we have compared the activity of promoters mainly active in macrophages, delineated as a macrophage expressing promoters. GFP gene as a quantitative reporter was used to evaluate the strength of promoters. Vectors were engineered with different promoters coding GFP readout for the study. pAcGFP-CMV has a strong CMV immediate-early promoter and was used as a positive control. Three (pAcGFP-MS, pAcGFP-EMR, pAcGFP-B5I) aforesaid promoter constructs with GFP reporter were compared. pAcGFP-NIX without promoter but with GFP gene was constructed as a negative control.

RAW264.7 cells (macrophage) and L929 (fibroblast) cells were selected for the study. These cell lines were selected to evaluate the behaviour of promoters in macrophage and non-macrophage cells respectively. Comparison of GFP expression through CMV promoter simultaneously in both the cells also helped us to analyze the difference in expression level due to difference in transfection efficiency.

To evaluate the activity of promoters under study, fluorescent microscopic analysis of GFP expressing cells were carried out. Fluorescence of GFP increased based on the expression which correlates to the activity of respective promoter. Besides the visual confirmation, functionality of all the promoter constructs was confirmed by Western blot of GFP which agreed to the microscopic analysis.

In order to assess the expression over large population of cells and achieve more sensitive data, flow cytometry was carried out for such differential expression. Mean fluorescent intensity (MFI) which was used for data acquisition is the average of certain number of cells obtained from individual cells in the population; such analysis provides the reproducible method to quantitate changes in reporter gene expression from a population. The expression of GFP by CMV promoter was robust in both the cells at all time points in comparison to other promoters (Figure 4). Among the macrophage specific promoter expression in RAW264.7, macrosialin showed higher expression followed by the other two constructs. Kinetics of promoter activity was assessed by evaluating reporter expression at various time points after transfection. All the constructs exhibited gradual increase in activity up to 24 hours, which decreased further. The expression of macrosialin promoter was significantly higher in macrophage cell line in comparison to non-macrophage cells. The ratio of macrophage/non-macrophage evaluation was the highest in macrosalin as an indicator of macrophage specificity. After 24 hours of analysis there was a decreasing trend in expression. The probable reason could be due to cells reaching confluence and underwent death, moreover the effect of toxicity of transfectant also increased over time. GFP fluorescence increased as per increase in protein concentration which was well depicted by Flow cytometry and fluorescent microscopy as reported earlier [43].

In order to understand the transcriptional activity of promoter, GFP mRNA levels were quantitated by one step qRT-PCR with TaqMan based probe chemistry developed in house. It supported the data obtained by Flow cytometry. Highest expression at all time point through CMV promoter was observed, followed by macrosialin. There was increasing trend in mRNA expression as per time, till 48 hours, however, after 72 hours no transcripts were detected. This difference may be attributed due to several reasons i.e. increase in level of toxicity over the time, there was no tight control over mRNA expression hence rigorous control over translation, all the mRNA were not turned into protein. The correlation at RNA and protein level depends upon the balance of transcriptional and translational regulatory elements [44]. The cessation of the expression of gene after limited expression could be beneficial for *in vivo *studies to avoid continued sustenance of antigen, since prolonged Ag exposure might lead to lower affinity Ab. Thus among the promoters under study, macrosialin directed the macrophage dominant expression in terms of both transcription and translation. Macrosialin governed the highest expression when compared with either EmrI or Beta-5 Integrin promoters. Regardless of the observed difference between mRNA or protein level, our finding clearly shows that macrosialin dominantly govern the expression in macrophage derived cells. It might be possible to use this promoter for directing expression of desired protein dominantly in APC.

Successful demonstration of APC dominant expression of GFP has opened an avenue to construct plasmids with virus encoded proteins. Use of these plasmids to evaluate the effect of cell dominant expression on the immune response and indication of protective ability would be interesting. In addition, targeting macrophage for various applications including immunotherapy might also be explored.

## Conclusions

To determine whether APC expressing promoters could be useful in terms of its specificity and activity, we compared with the CMV immediate early promoter in macrophage and non-macrophage derived cells. The activity of macrosialin was significantly higher in macrophage cells in comparison to EmrI and Beta-5 Integrin, whereas CMV showed the highest activity in both the cell types. Our work presents a systematic *ex vivo *study at the level of protein expression and mRNA transcription. This indicates that macrosialin promoter might prove beneficial for targeting expression majorly in APC, however *in vivo *potential needs to be carried out for its suitable application.

## Competing interests

The authors declare that they have no competing interests.

## Authors' contributions

MFA has planned, designed and carried out all the experiments. MMG envisioned and supervised all the studies. Both the authors read and approved the final manuscript.
